# Editorial: Best Papers of 2009

**DOI:** 10.1120/jacmp.v11i3.3397

**Published:** 2010-06-22

For the past several years, the JACMP has issued awards to several manuscripts deemed by the Board of Editors to be worthy of the designation “Best Papers.” These awards are selected by a committee of the Board of Editors and are supported by grants from some of our corporate sponsors. By listing the Best Papers of 2009, I would like to honor the award winners for their contributions to the clinical practice of medical physics and thank our corporate sponsors for their financial support.

- The RIT Award of Excellence for the Best Medical Imaging Article was awarded to Joanna R. Fair, Philip H. Heintz, and Robert J. Telepak, “Evaluation of new data processing algorithms for planar gated ventriculography (MUGA),” JACMP 10(3):173–179.
- The Sun Nuclear Award of Excellence for the Best Radiation Oncology Physics article was awarded to Joseph P. Santoro, Ellen Yorke, Karyn A. Goodman, and Gig S. Mageras, “From phase‐based to displacement‐based gating: a software tool to facilitate respiration‐gated radiation treatment,” JACMP 10(4):132–141.
- The Unfors Award of Excellence for the Best Radiation Measurements article was awarded to Danielle Fraser, William Parker, and Jan Seuntjens, “Characterization of cylindrical ionization chambers for patient specific IMRT QA,” JACMP 10(4):241–251.
- The LAP Award of Excellence for Outstanding Radiation Oncology Physics article was awarded to Eduard Schreibmann, Eric Elder, and Tim Fox, “Automated Quality assurance for image‐guided radiation therapy,” JACMP 10(1):71–79.
- The Varian Editor‐in‐Chief Award of Excellence for the Best General Medical Physics article is awarded to R. Alfredo Siochi, Edward C. Pennington, Timothy J. Waldron, and John E. Bayouth, “Radiation therapy plan checks in a paperless clinic,” JACMP 10(1):43–62.

Congratulations to all the award winners, and thanks very much to all the award sponsors.

George Starkschall, PhD

Editor‐in‐Chief

August 15, 2010

